# Nosocomial Neonatal Legionellosis Associated with Water in Infant Formula, Taiwan

**DOI:** 10.3201/eid2011.140542

**Published:** 2014-11

**Authors:** Sung-Hsi Wei, Pesus Chou, Lei-Ron Tseng, Hung-Chih Lin, Jen-Hsien Wang, Ji-Nan Sheu, Ming-Tsan Liu, Fang-Ching Liu, Hoa-Hsin Wu, Min-Cheng Lin, Ching-Fen Ko, Hsiang-Yu Lin, Pei-Hsiu Kao, Kao-Pin Hwang, Yu-Lung Hsu, Tsung-Lin Kuo, Chuen-Sheue Chiang

**Affiliations:** Centers for Disease Control, Taipei, Taiwan (S.-H. Wei, L.-R. Tseng, M.-T. Liu, H.-H. Wu, M.-C. Lin, C.-F. Ko, P.-H. Kao, T.-L. Kuo, C.-S. Chiang);; Community Medicine Research Center and Institute of Public Health, National Yang-Ming University, Taipei, Taiwan (S.-H. Wei, P. Chou);; China Medical University Hospital, Taichung, Taiwan (H.-C. Lin, J.-H. Wang, H.-Y. Lin, K.-P. Hwang, Y.-L. Hsu);; School of Medicine, Chung Shan Medical University and Hospital, Taichung (J.-N. Sheu); Jen-Ai Hospital, Taichung (F.-C. Liu);; National Taipei University of Nursing and Health Sciences, Taipei (C.-S. Chiang)

**Keywords:** water, infant formula, *Legionella*, neonatal legionellosis, neonate, nosocomial infection, Taiwan, bacteria

## Abstract

We report 2 cases of neonatal *Legionella* infection associated with aspiration of contaminated water used in hospitals to make infant formula. The molecular profiles of *Legionella* strains isolated from samples from the infants and from water dispensers were indistinguishable. Our report highlights the need to consider nosocomial legionellosis among neonates who have respiratory symptoms.

*Legionella* infection was first reported in adults at an American Legion convention in Philadelphia in 1976 (*1*). The disease has a variety of clinical manifestations, ranging from mild respiratory tract illness to fatal pneumonia, especially among immunocompromised persons (*2*).

*Legionella* infection in neonates occurs rarely among both healthy and immunocompromised patients (*3**–**8*). Water birth and use of cold-mist humidification have been associated with neonatal legionellosis (*9**–**11*), but investigations of transmission modes are limited. We report 2 cases of neonatal *Legionella* infection associated with contaminated water used in infant formula in a hospital setting.

## The Cases

Case 1 involved a male neonate delivered by cesarean section in obstetric hospital A in Taiwan in April 2013, after an uneventful pregnancy of 38 weeks. He was fed infant formula in the hospital’s nursery. On postpartum day 7, he had a fever of 39°C and tachypnea and was taken to a tertiary hospital. Chest radiograph showed a ground-glass opacity in the middle lobe of his right lung, which subsequently developed into cavities. Microbiological testing of sputum and blood specimens yielded no definitive results. His pulmonary condition deteriorated during the following days. The case was reported to the Unknown Pathogen Discovery/Investigation Group at the Taiwan Centers for Disease Control for extensive etiologic survey. Multiplex real-time reverse transcription PCR showed sputum and blood specimens to be negative for adenovirus, respiratory syncytial virus, coronaviruses (229E, OC43, NL63, HKU1, and middle east respiratory syndrome coronavirus), metapneumovirus, influenza virus, parainfluenza viruses 1–4, herpes simplex viruses 1 and 2, varicella-zoster virus, Epstein-Barr virus, cytomegalovirus, human herpesviruses 6 and 7, bocavirus, parvovirus, enterovirus, and rhinovirus. However, *Legionella pneumophila* serogroup 5 was isolated from the sputum specimen. The patient was treated with antibacterial drugs and was discharged on postpartum day 17.

Water specimens from all sources with which the patient had contact were tested for *Legionella* by culture. *L. pneumophila* serogroup 4 was isolated from 3 tap water sources, and *L. pneumophila* serogroup 5 was isolated from the cold water source of the hot and cold water dispenser from which water for making infant formula was collected (Table). The water dispenser was located in the room next to the nursery. The hot water faucet provided boiled water at 95°­100°C, and the cold water faucet provided unboiled water that was treated by a built-in reverse osmosis device. The pulsed-field gel electrophoresis (PFGE) profiles of the *L. pneumophila* serogroup 5 strains isolated from a sputum sample from the infant and those from a water sample from the water dispenser were indistinguishable (Figure). Both strains were sequence type 1032 (*12*).

**Table:** Results of *Legionella* culture in environmental water specimens associated with nosocomial infection of neonates, Taiwan*

Site of sampling		1st Sampling,† strain (sequence type), concentration (CFU/L)‡			2nd Sampling,† strain (sequence type), concentration (CFU/L)	
Case 1						
Tap water source 1 for bathing infants in nursery	*L. pneumophila* serogroup 4 (ST1712), 2.5x10^3^			*L. pneumophila* serogroup 4 (ST1712), TMTC		
Tap water source 2 in nursery	*L. pneumophila* serogroup 4 (ST1712), 2x10^3^			*L. pneumophila* serogroup 4 (ST1712), TMTC		
Drainage from air conditioner in nursery	ND			Negative		
Tap water source in room adjacent to nursery	ND			*L. pneumophila* serogroup 4 (ST1764), TMTC		
Cold water source of the hot/cold water dispenser§	*L. pneumophila* serogroup 5 (ST1032)¶, 2.2x10^4^			ND		
Tap water source in maternity ward	ND			Negative		
Case 2						
Tap water source for rinsing infants in nursery	Negative			Negative		
Tap water source for reverse osmosis water in nursery	Negative			Negative		
Cold water source of the hot/cold water dispenser§	*L. pneumophila* serogroup 1 (ST1)¶, 2x10^2^			*L. pneumophila* serogroup 1 (ST1)¶, TMTC		
Hot water source of the hot/cold water dispenser	ND			Negative		
Tap water source 1 in maternity ward	Negative			Negative		
Tap water source 2 in maternity ward	ND			Negative		
Tap water source 3 in maternity ward	Negative			Negative		
Tap water source 4 in maternity ward	ND			Negative		
Tap water source for bathing in infant’s house	ND			Negative		
Tap water source for drinking water in infant’s home	ND			Negative		

*Both case-patients were delivered by cesarean section and were not breast fed or placed in incubators. All potential infection sources were tested and listed. No humidifier was used in either nursery. ST, sequence type; TMTC, too many to count; ND, testing not done. †The first samples from the environments of case-patients 1 and 2 were collected 33 d and 23 d after birth, respectively. The second samples were collected 34 d and 26 d after birth, respectively. ‡Concentration of *Legionella* spp. detected in water specimens, attributed to the presence of sampling swabs. §Used in formula for neonates. ¶The pulsed-field gel electrophoresis profile of the strain was indistinguishable from that of the strain isolated from the patient’s sputum. The ST of the strain was the same as that of the strain isolated from the patient’s sputum.

**Figure:**
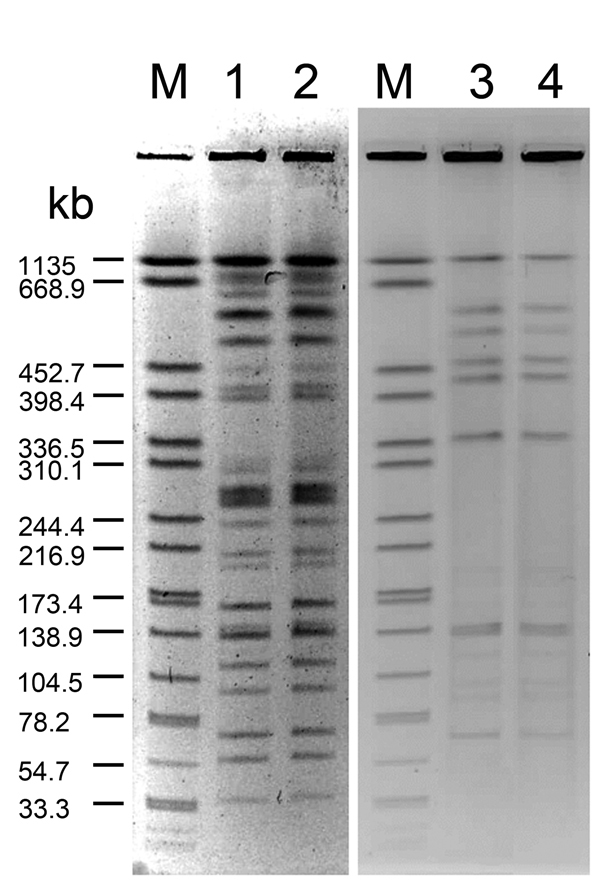
Pulsed-field gel electrophoresis patterns for *Legionella pneumophila* isolates from neonates with *Legionella* infection and hospital water sources. Genomic DNA was digested with *Sfi*I and separated in 1% agarose gel by Bio-Rad CHEF MAPPER. Lane M, reference size maker (*Xba*I-digested genomic DNA fragments of *Salmonella enterica* ser. Braenderup H9812); lanes 1 and 2, clinical and environmental isolates of *L. pneumophila* serogroup 5 from case-patient 1; lanes 3 and 4, clinical and environmental isolates of *L. pneumophila* serogroup 1 for case-patient 2.

Parents of the 175 neonates that were delivered ≤3 months before the patient’s birth in hospital A were interviewed by telephone; 3 of the 175 infants had fever within the first month of life. Serologic testing 3 weeks after their fever episodes was negative for IgM or IgG antibodies against *L. pneumophila* serogroups 1–6.

The water dispenser in hospital A was replaced with one that provided only hot water. The point-of-use filters were mounted on the water faucets in the nursery (Pall-AquaSafe Water Filter, Portsmouth, UK). All subsequent tests of water samples were negative for *Legionella* spp. No *Legionella* infection was identified in any neonate who was born after the patient’s birth in hospital A during the following 8 months.

Case 2 involved an asymptomatic male neonate delivered by cesarean section in obstetric hospital B in November 2013 after an uneventful pregnancy of 38 weeks. An optional screening test requested by the parents for severe combined immunodeficiency was negative. He remained asymptomatic and was fed infant formula throughout his 8-day stay in the hospital. At home on the day of discharge, he had a fever of 38.5°C and poor appetite. He had tachypnea and cyanosis of the lips during the following days, and on day 10 after birth, he was taken to the same tertiary hospital as case-patient 1. Bilateral pulmonary infiltrates and mild right pneumothorax were identified. The patient’s urine and sputum specimens tested positive for *L. pneumophila* serogroup 1. Azithromycin was administered on the day of admission. His clinical condition improved gradually. However, pulmonary fibrosis and pneumatoceles were identified later. He was discharged on day 55 after birth.

Testing of related environmental water specimens for *Legionella* spp. by culture was negative for all specimens except that from the cold water source of the hot and cold water dispenser in hospital B , which was positive for *L. pneumophila* serogroup 1 (Table). The water dispenser in the nursery, which consisted of a tank containing boiled, hot water and another tank containing cool water pipelined from the boiled water tank, was used for making infant formula. The PFGE profiles of the strains from the patient and those from the water dispenser were indistinguishable (Figure). Both strains were sequence type 1.

The parents of the 79 neonates born ≤3 months before the patient’s birth in hospital B were interviewed by telephone. None of the neonates had fever during the first month of life. The water dispenser in hospital B was replaced with one that provided only hot water. All following water tests showed negative results. No *Legionella* infection was identified in any neonate who was born after the patient’s birth in hospital B during the following 3 months.

## Conclusion

We report 2 cases of neonatal legionellosis associated with infant formula prepared with contaminated water. Only the cold water from both hospitals’ hot and cold water dispensers used for making infant formula was positive for *L. pneumophila*, with indistinguishable PFGE profiles and the same sequence types as those isolated from the neonates. Some environmental specimens were tested twice to compensate for inadequate sensitivity of the culture method and yielded the same results. No humidifier was used in either case. In the hospital where the first case-patient was identified, the water dispenser was not located in the nursery, which likely reduced the risk of transmitting *Legionella* spp. through contaminated aerosol droplets generated by the water dispenser.

Aspiration is a major transmission mode in hospital-acquired *Legionella* infection among adults (*13**,**14*). Neonatal *Legionella* infection through aspiration of contaminated water during water birth has been reported (*9**,**10*). Our results also indicate the *Legionella* infection described here was likely transmitted by aspiration of contaminated infant formula during the first days of life.

Both water dispensers were colonized with *Legionella* spp. Although the pervasiveness of these 2 problematic types of water dispenser is not known, hot and cold water dispensers are used in many hospitals in Taiwan. We speculate the complicated pipeline system and inappropriate maintenance of water dispensers might increase the risk for *Legionella* colonization, thus facilitating *Legionella* infection. Our report highlights the potential risk for *Legionella* transmission posed by contaminated water dispensers.

Few cases of neonatal legionellosis have been reported in the literature (*3*–*5*). Most cases were hospital acquired, and the patients had severe pneumonia that was associated with a high mortality rate (*8**,**15*). The manifestation of *Legionella* infection is not pathognomonic (*15*), therefore, underdiagnoses or delayed diagnoses combined with inappropriate treatment likely contribute substantially to mortality rates and severity of neonatal legionellosis. The 2 neonates described here survived after prompt diagnosis and treatment enabled by an extensive etiologic investigation and high suspicion of *Legionella* infection. Our report underscores the importance of suspecting and testing for *Legionella* infection when neonates have respiratory symptoms.
